# Resource Characteristics of Common Reed (*Phragmites australis*) in the Syr Darya Delta, Kazakhstan, by Means of Remote Sensing and Random Forest

**DOI:** 10.3390/plants14060933

**Published:** 2025-03-16

**Authors:** Azim Baibagyssov, Anja Magiera, Niels Thevs, Rainer Waldhardt

**Affiliations:** 1 International Ph.D. Program in Agricultural Economics, Bioeconomy and Sustainable Food Systems (IPPAE), Justus Liebig University Giessen, Senckenbergstrasse 3, 35390 Giessen, Germany; 2 Division of Landscape Ecology and Landscape Planning, Institute of Landscape Ecology and Resources Management, Center for International Development and Environmental Research (ZEU), Justus Liebig University Giessen, 35390 Giessen, Germany; anja.magiera@agrar.uni-giessen.de (A.M.); rainer.waldhardt@umwelt.uni-giessen.de (R.W.); 3 Gesellschaft Für Internationale Zusammenarbeit (GIZ), Gluckstraße 2, 53115 Bonn, Germany; niels.thevs@gmail.com

**Keywords:** common reed, inland wetland mapping, reed beds, standing biomass assessment, remote sensing, multiple random forest regression models, Syr Darya Delta

## Abstract

Reed beds, often referred to as dense, nearly monotonous extensive stands of common reed (*Phragmites australis*), are the most productive vegetation form of inland waters in Central Asia and exhibit great potential for biomass production in such a dryland setting. With its vast delta regions, Kazakhstan has the most extensive reed stands globally, providing a valuable case for studying the potential of reed beds for the bioeconomy. However, accurate and up-to-date figures on available reed biomass remain poorly documented due to data inadequacies in national statistics and challenges in measuring and monitoring it over large and remote areas. To address this gap in knowledge, in this study, the biomass resource characteristics of common reed were estimated for one of the significant reed bed areas of Kazakhstan, the Syr Darya Delta, using ground-truth field-sampled data as the dependent variable and high-resolution Sentinel-2 spectral bands and computed spectral indices as independent variables in multiple Random Forest (RF) regression models. An analysis of the spatially detailed yield map obtained for *Phragmites australis*-dominated wetlands revealed an area of 58,935 ha under dense non-submerged and submerged reed beds (with a standing biomass of >10.5 t ha^−1^) and an estimated 1,240,789 tons of reed biomass resources within the Syr Darya Delta wetlands. Our findings indicate that submerged dense reed exhibited the highest biomass at 28.21 t ha^−1^, followed by dense non-submerged reed at 15.24 t ha^−1^ and open reed at 4.36 t ha^−1^. The RF regression models demonstrated robust performance during both calibration and validation phases, as evaluated by statistical accuracy metrics using ten-fold cross-validation. Out of the 48 RF models developed, those utilizing the Normalized Difference Vegetation Index (NDVI) and Normalized Difference Water Index (NDWI) as key predictors yielded the best standing reed biomass estimation results, achieving a predictive accuracy of R^2^ = 0.93, Root Mean Square Error (RMSE) = 2.74 t ha^−1^ during the calibration, and R^2^ = 0.83, RMSE = 3.71 t ha^−1^ in the validation, respectively. This study highlights the considerable biomass potential of reed in the region’s wetlands and demonstrates the effectiveness of the RF regression modeling and high-resolution Sentinel-2 data for mapping and quantifying above-ground and above-water biomass of *Phragmites australis*-dominated wetlands over a large extent. The results provide critical insights for managing and conserving wetland ecosystems and facilitate the sustainable use of *Phragmites australis* resources in the region.

## 1. Introduction

Inland wetlands rank among the most biologically productive ecosystems in the world and are home to some large aquatic plants, also known as macrophytes [1,2,3,4]. These plants, adapted to grow partially or fully submerged in water, significantly enhance the ecological complexity and functionality of these vital habitats while providing substantial ecological, economic, and social benefits [5,6,7].

One prominent example of such aquatic plant species worldwide is the common reed (*Phragmites australis*) [8,9]. This tall, perennial emergent graminoid is recognized for its high net primary productivity and extensive global distribution. Typically classified as a hydrophyte or helophyte, it flourishes in waterlogged and inundated areas with little competition from other vegetation. As a result, it often dominates the aquatic flora of inland waters across diverse climatic zones and geographical regions, forming extensive, nearly homogeneous stands in deltas, along lakeshores, and in almost any natural or man-made marshy area [10,11].

Although these characteristics position common reed as a key species in various aquatic and freshwater ecosystems worldwide, its role within natural communities is perceived controversially [12]. Depending on the social and ecological context in which common reed and its ecosystem services are embedded, it is regarded as beneficial for wetland ecosystems or an invasive species [13]. For instance, in some areas of North America, its rapid spread has been identified as detrimental to native species and overall wetland biodiversity; hence, management measures have been taken to curb its spread [14,15,16]. In other regions, however, it is recognized for its multifunctional roles within wetland ecosystems, such as providing habitats for a number of other species and as a source of biomass, highlighting its capacity to serve as a feedstock source for the bio-based circular economy and categorizing it among the promising sources of renewable biomass worldwide [17,18,19]. Moreover, its high levels of peat formation make common reed the most suitable and frequently used wetland plant for paludiculture and phytoremediation [20,21,22]. Therefore, common reed has the potential to be an aquatic plant that provides a number of benefits [23].

The vast areas of reed beds across the wetlands of Central Asia make this region particularly relevant to exploring the benefits of this aquatic plant [24,25]. It produces huge amounts of lignocellulosic biomass, representing a significant albeit often overlooked and underestimated resource that can be tapped as a raw material for the bioeconomy, even if only some of these reeds are used to ensure space for biodiversity conservation [26,27]. Currently, small quantities of reed are traded internationally in the form of thatch. But, with the growing global demand for sustainably sourced biomass [28], these vast reed biomass resources could develop into a valuable domestic source of feedstock for a bio-based circular economy and generate economic opportunities for rural areas [26,27]. Though this potential is receiving increasing attention from local stakeholders and policymakers, fine-scale national inventories of a spatially detailed assessment of common reed’s above-ground biomass, which serve as a basis for sound regional land-use planning, including reed biomass sourcing, are lacking across the whole region of Central Asia. Therefore, accurate and cost-effective methods for estimating above-ground and above-water standing reed biomass are essential for successfully establishing and maintaining reed stocks for the bioeconomy while preventing overutilization. In addition to biomass, reed stands provide various ecosystem services, including animal fodder provision, water cycle regulation, and recreational opportunities [24,25,29,30].

Kazakhstan is home to the world’s most extensive reed bed areas, accounting for 20% of the total global expanse [23]. This is mainly attributed to its extensive wetland complexes in the river deltas and along inland water bodies [26]. Despite this valuable opportunity to explore the potential of reed beds for the bioeconomy, assessments of the area and distribution of reed beds, as well as management advice in these delta regions, remain limited. While a detailed assessment and mapping of wetlands area and reed biomass resources in the Ili Delta at Lake Balkhash have been previously conducted [24], similar studies for other major reed bed areas in the country—such as the Syr Darya Delta at the Lesser Aral Sea, the Ural Delta at the Caspian Sea and similar—have yet to be performed. Consequently, reliable and up-to-date information regarding the area, the spatial distribution of reed beds, and their biomass potential in these areas is still missing, even though further research into the utilization of reed beds and their integration into landscape planning in Kazakhstan primarily focuses on these deltas.

In light of this background, the objectives of this study were to model a spatially explicit yield map for *Phragmites australis*-dominated wetlands and contribute to closing the gap of missing reed distribution and biomass data by quantifying the area and standing biomass of reed beds in the Syr Darya Delta, Kazakhstan. The wetland ecosystems within the Syr Darya Delta near the Aral Sea, including the areas of abandoned cropland now covered by reed of the adjacent Qazaly Irrigation Zone, were selected as a model case study for conducting such a mapping exercise. Hence, this study may serve as a blueprint for further mapping efforts of reed areas and biomass in analogous contexts and contribute to building a better knowledge base on submerged plants and options for their utilization, thereby facilitating informed planning and management.

To guide this research, two key questions motivated the study: (1) What is the area and spatial distribution of wetlands and *Phragmites australis*-dominated vegetation in the Syr Darya Delta, divided into submerged and non-submerged reed beds? (2) Building on the preceding, what is the standing biomass of *Phragmites australis* in the Syr Darya Delta? Here, the term “standing biomass” thereby refers to fresh biomass and excludes all dead biomass from previous years.

To address these research questions, this paper presents, for the first time, a spatially explicit yield map of *Phragmites australis*-dominated wetlands in the Syr Darya Delta, Kazakhstan utilizing remote sensing and Random Forest (hereafter “RF” or “rf”) [31] regression modeling to assess their area and biomass potential. The application of data from the Sentinel-2 satellite, with its high-resolution imagery (10 m × 10 m) and frequent revisiting times of 3–5 days, is particularly suited for monitoring dynamic ecosystems like wetlands that rely on fluctuating water supplies. By comparing the standing biomass in the Syr Darya Delta to that in the Ili Delta and other regions, as well as to traditional biomass resources such as wood, we report on the considerable biomass potential of common reed in the region’s wetlands, emphasizing its ecological and socio-economic significance as a sustainable feedstock source for the bioeconomy, particularly in arid regions like Central Asia.

## 2. Results

### 2.1. Plot-Level Above-Ground and Above-Water Surface Standing Biomass of Reed Beds and Relationships with Remote Sensing Variables

**Figure 1:**
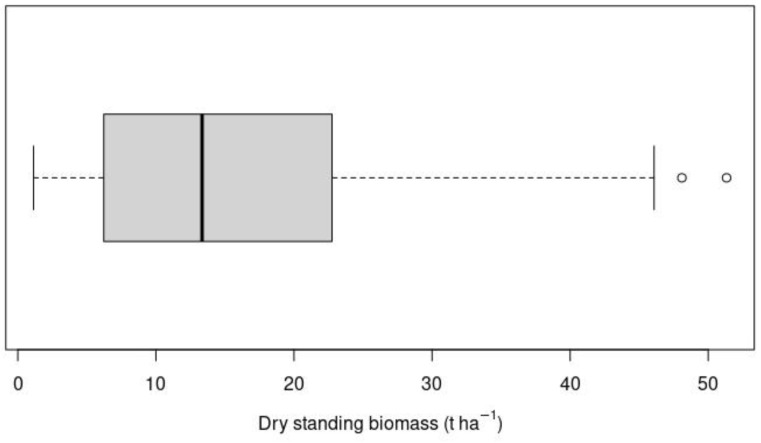
Box–whisker plot of the *Phragmites* biomass based on plot-level data (*n* = 78) collected in the Syr Darya Delta, Kazakhstan, during the 2019–2020 period. Whiskers represent the minimum and maximum of the data, while the circles represent outliers, which are outside 1.5 times the interquartile range. The box boundaries are the first and third quartiles, and the black line is the median value.

The dry standing above-ground and above-water surface biomass of reed beds (hereafter “*Phragmites* biomass” or “reed biomass”) ranged from 1.14 t ha^−1^ to 51.33 t ha^−1^, with a mean biomass of 15.94 t ha^−1^ (Figure 1).

A comparative analysis of reed biomass across the various land cover classes revealed that the land cover class with the submerged dense reed exhibited the highest mean biomass at 28.21 t ha^−1^. This was followed by the land cover class of the dense non-submerged reed with a mean biomass of 15.24 t ha^−1^, while the land cover class of the open reed and shrub vegetation had a significantly lower mean biomass of 4.36 t ha^−1^, as summarized in Table 1. Notably, the maximum *Phragmites* biomass of 51.33 t ha^−1^ was recorded within the land cover class with the submerged dense reed.

An examination of correlation coefficients, conducted prior to constructing the spatially detailed yield assessment models using the Random Forest machine learning algorithm, revealed considerable variability in the strength of the correlation between the above-ground and above-water standing biomass of reed beds (referred to as biomass) and the spectral data obtained from the Sentinel 2 satellite, which served as explanatory variables in the regression models.

With an R^2^ of 0.72, the NDVI exhibited a robust positive correlation with the standing biomass values of reed beds, followed by the NIR, with an insignificant positive relationship. The rest, conversely, displayed a negative correlation with the NDWI with an R^2^ of −0.64, significantly leading and suggesting an inverse relationship with the standing biomass.

### 2.2. The Random Forest Model Performance and Mapping of Above-Ground and Above-Water Surface Reed Biomass and Spatial Distribution Patterns

The R^2^ values ranged from 0.34 to 0.81, while the RMSE values ranged from 4.08 t ha^−1^ to 10.93 t ha^−1^ (see Appendix A for additional details).

**Figure 2:**
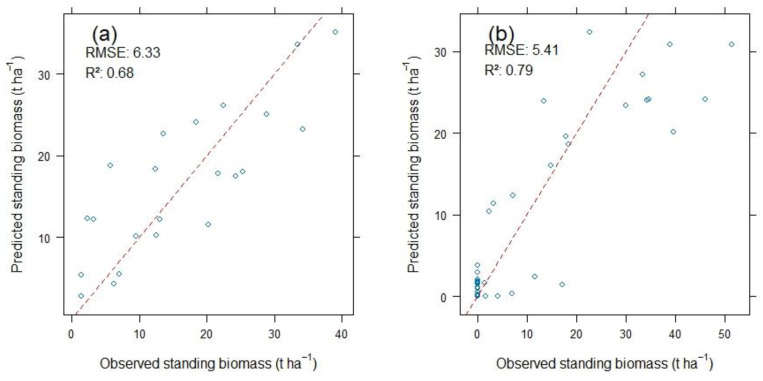
Scatter plots illustrating the relationship between predicted and observed standing biomass values of reed in the Syr Darya Delta, Kazakhstan, within the testing (validation) dataset: (**a**) by the rf37 model, solely based on reed biomass data (n = 78), and (**b**) by the rf41 model, which incorporates both reed biomass data and land cover data with no biomass (n = 205).

Among the 24 RF regression models developed based on reed biomass data alone, the rf37 model (illustrated in Figure 2a) showed the highest predictive accuracy, with an RMSE of 6.81 t ha^−1^ and an R^2^ of 0.77 during calibration. After validation, it maintained an RMSE of 6.33 t ha^−1^ and an R^2^ of 0.68. Conversely, among the other 24 RF regression models that incorporated both reed biomass and land cover data, the rf41 model (shown in Figure 2b) demonstrated superior performance, achieving the lowest RMSE of 4.08 t ha^−1^ and the highest R^2^ of 0.81 during calibration, followed by an RMSE of 5.41 t ha^−1^ and an R^2^ of 0.79 during validation. The performance of these models in estimating standing reed biomass, as evaluated on the testing (validation) dataset during model validation, is depicted in the scatter plots comparing the predicted standing biomass to the observed standing biomass of the validation dataset (Figure 2).

**Figure 3:**
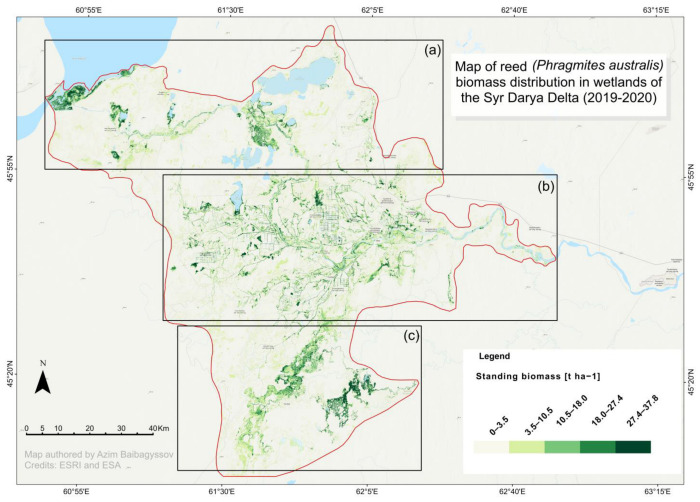
The Random Forest regression-predicted map showcasing the standing biomass of *Phragmites australis* (in t ha^−1^) across three investigation areas of the Syr Darya Delta, Kazakhstan, in 2019–2020. WGS 84/UTM zone 41 N (EPSG: 32641). (**a**) Wetland areas adjacent to the Kok-Aral dam and dike complex; (**b**) wetland areas around deltaic lakes, such as Aidarkol and Kotankol next to the Bekarystan Bi Village; and (**c**) wetland areas along the left branch of the Syr Darya River next to Tasaryk and Lakaly Villages and around the Maryamkol Lake next to the Kaukei Village. Beige color corresponds to areas with predicted low (0–3.5 t ha^−1^) standing reed biomass, while gradient greens correspond to areas predicted to harbor intermediate (3.5–10.5; 10.5–18.0 t ha^−1^) and high (18.0–27.4; 27.4–37.8 t ha^−1^) reed biomass classes.

Considering these model performance results, a final map (WGS 84/UTM zone 41 N) (Figure 3) illustrating the spatial distribution of above-ground and above-water surface *Phragmites* biomass in the Syr Darya Delta, Kazakhstan, was produced using the model of the best fit, i.e., the rf41 model. Performed in the Forest-Based Classification and the Regression Tool of ArcGIS Pro 3.2.2, this mapping yielded a slightly higher predictive accuracy with an R^2^ = 0.93 and a lower RMSE = 2.74 t ha^−1^ during the model calibration, along with an R^2^ = 0.83 and an RMSE = 3.71 t ha^−1^ at its validation. The slightly better outputs can be attributed to ArcGIS Pro’s advanced geospatial analysis capabilities and optimized algorithms to handle spatial data more effectively.

This resulting map clearly delineates the spatial distribution of the above-ground and above-water surface standing reed biomass at the landscape level within the Syr Darya Delta, Kazakhstan. According to its spatial distribution patterns, dense standing *Phragmites* biomass is predominantly located along the Syr Darya River’s right (Figure 3a) and left (Figure 3c) branches and along the shores of deltaic lakes. These lakes are distributed in the northern, northwestern, southern, and southeastern parts of the Syr Darya Delta, Kazakhstan. In contrast, the central part of the delta showcases lower biomass densities as it is primarily occupied by irrigated croplands (Figure 3b). In this area, reed growth is concentrated mainly along irrigation canals and in abandoned as well as post-flooding lands unsuitable for regular cropping.

### 2.3. Reed Bed Area Assessment and Biomass Resource Quantification

The area of each of the classes from Figure 3 is listed in Table 2.

The total above-ground and above-water surface biomass of *Phragmites australis* within the study area for 2019–2020 was estimated at approximately 2.5 million tons. A significant portion of the study area’s land, accounting for 75.4%, falls within a broad land cover class that includes open water, bare land with open sands, and areas considered to be open reed and shrub vegetation. This is followed by the land cover class with non-submerged dense reed beds (18.9%) and the land cover class with submerged dense reed beds (5.7%).

The combined area of land cover classes of dense non-submerged and submerged reed beds—exhibiting standing biomass greater than 10.5 t ha^−1^—totaled 58,935 hectares. This area translates to an estimated 1,240,789 tons of reed biomass. These considerable biomass resource figures highlight the abundant availability of *Phragmites* biomass within the wetlands of the Syr Darya Delta. Furthermore, they underscore the significant economic potential of this resource for the sustainable livelihoods of the local population, particularly in its prospective role as a domestic feedstock for sustainable biomass utilization in the region.

## 3. Discussion

### 3.1. Estimated Reed Biomass and Its Spatial Distribution in the Syr Darya Delta, Kazakhstan

The *Phragmites* biomass of the submerged dense reed class in this study is in the same range as the *Phragmites* biomass measured in Lake Burullus in Egypt, 54 t/ha, as reported by [32], the Ili Delta in Kazakhstan, 5.5 to 57.2 t/ha according to [24], or further studies listed by [33]. The reed biomass values of the land cover classes non-submerged dense reed and open reed and shrub vegetation of this study are higher than the corresponding values from the Ili Delta [24]. This difference might be explained by a higher grazing pressure observed in the Ili Delta compared to the study area at the Syr Darya, as the two non-submerged reed land cover classes in the Ili Delta are frequently grazed. Such grazed areas show a lower NDVI and, thus, lower biomass compared to non-grazed areas.

The spatial distribution of the reed area with high biomass is particularly pronounced along river branches, irrigation canals, and deltaic lake shores. This pattern mirrors observations made in other deltas, such as the Ili Delta, and indicates that hydrological factors play a vital role in determining reed biomass distribution in these continental arid regions. This is further supported by the relationship between reed biomass and NDWI, which is the second most important variable across all models in this context. Water bodies create optimal conditions for reed growth, as they are regularly inundated and maintain consistent moisture throughout the growing season [34,35].

In contrast, the desiccation and drying of constricted and small deltaic lakes adversely impact the growth and viability of reed beds, leading to their localized thinning, a reduction in extent due to an elevation of extremely unfavorable salinity in both water and soil, and drying of the soil [33,36].

Moreover, the productivity of natural reed beds is shaped not only by the ecological features of their environment but also the genetic variation and phenotypic traits of local ecotypes, as discussed by [37,38]. While [36] suggested that the phenotypes of *Phragmites australis* in continental arid environments align with flood regimes and topsoil salinization, [33] reported substantial variability in productivity among different *Phragmites australis* phenotypes in relation to their specific growth conditions and grazing pressures identified in Southern Xinjiang. Echoing these findings, varying phenotypes of *Phragmites australis* have also been observed within this study in the Syr Darya Delta; however, the genetic variation patterns across these populations within this arid region of Central Asia and their implications for productivity remain unexplored, highlighting a critical area for further research.

### 3.2. Implications for Management Strategies and Sustainable Use of Reed Biomass Resources

The biomass that can be harvested annually from the submerged reed as raw material is substantially higher than from tree plantations or forests, e.g., *Populus* and *Picea schrenkiana*, which are the most important timber and fuel wood species in the southern part of Kazakhstan. Shatalov (1973) [39] reports a volume stock of 465 m^3^/ha for a 30-year-old *Populus nigra* stand in the steppe zone of Kazakhstan. This corresponds to an average annual growth of 5.47 t/ha (considering a wood density of 0.353 g/cm^3^ according to Chave et al. [40]). Notably, the reed biomass of the submerged reed in this study is in the same range of the most optimistic assumption for the yield of a poplar plantation with modern poplar cultivars [41], thereby underscoring the potential of the reed as a viable biomass resource.

Similarly, for a 60-year-old *P. schrenkiana* stand, Kozlovskiy and Pavlov [42] listed a volume stock of 374 m^3^/ha, which corresponds to an average annual increment of 2.26 t/ha, again considering a wood density of 0.362 g/cm^3^ [40]. These comparisons emphasize the remarkable biomass potential of submerged reed in the region, which may yield substantial ecological and economic benefits through improved harvesting and utilization practices.

The strategic harvesting and utilization of reed biomass present a dual opportunity: it could substantially reduce the country’s reliance on imported wood and lower pressure on forests across Kazakhstan. By incorporating reed biomass into various industrial applications, such as the production of chipboards—a widely used material for residential construction across Central Asia—stakeholders could create a sustainable alternative that fosters local economic development while promoting the conservation and wise use of wetland and forest ecosystems [24,43].

Furthermore, establishing a market for reed biomass not only aids in environmental conservation but also contributes to the bioeconomy, particularly in underdeveloped rural areas such as the downstream regions of Kazakhstan [26]. Consequently, it is essential for policymakers to formulate management strategies that prioritize research, incentives for sustainable harvesting, and technological advancements in biomass processing. Through these initiatives, Kazakhstan can pave the way for an eco-efficient approach to resource utilization that aligns with global sustainability goals. Ultimately, the recognition of reed biomass as a primary resource can facilitate a more harmonious balance between economic development and ecological stewardship in the region.

### 3.3. Limitations of Above-Ground and Above-Water Surface Wetland Biomass Assessment Using Random Forest Predictive Modeling and Satellite Data

In recent years, there has been growing scientific interest in using the Random Forest machine learning algorithm and remote sensing techniques to estimate above-ground biomass in various ecosystems. Significant improvements in the mapping and assessment of above-ground biomass have been reported for ecosystems such as forests [44,45,46], including mangroves [47,48], grasslands [49,50,51], rangelands [52,53], savannas and woodlands [54,55,56,57], and wetlands [58,59,60]. Likewise, this study demonstrates the effectiveness and robustness of the Random Forest regression model in combination with high-resolution Sentinel-2 remote sensing data and ground-truth data in mapping and quantifying *Phragmites* biomass within the wetland ecosystems of the Syr Darya Delta, Kazakhstan.

The Normalized Difference Vegetation Index (NDVI) showed a strong positive correlation with the standing biomass of reed beds, confirming its utility as a reliable predictor for biomass. In contrast, the Normalized Difference Water Index (NDWI) exhibited a significant inverse correlation, indicating an alternative ecological dynamic related to water content and biomass levels. However, consistent with findings from other biomass assessment studies [58,61,62], we observed an asymptotic saturation trend in the NDVI as *Phragmites* biomass increased. Due to this phenomenon, the predictive accuracy of the Random Forest regression model is limited, particularly when the observed biomass exceeds 30 t ha^−1^ in areas with dense submerged stands.

Despite the practicality of the Random Forest regression method, several challenges and limitations are often encountered during its application to map and assess wetland above-ground and above-water biomass. For instance, the complexity of spectral signatures in wetland environments—due to the presence of water, shadowing, and varying vegetation densities—makes it challenging to accurately differentiate and quantify above-ground and above-water biomass [63,64]. Additionally, validating biomass estimation models derived from the Random Forest algorithm and high-resolution Sentinel-2 data requires comprehensive and robust ground-truth data collection; uncertainties linked to field measurements may affect above-ground biomass inversion due to spatial and temporal variability in plant community characteristics and seasonal productivity, such as vegetation phenology [62,65,66].

Discrepancies may arise from sampling errors, variations in the spatial resolution of biomass sampling sites in relation to the resolution of raster data, or differences in the timing of data collection compared to satellite overpass, all of which can introduce spatial and seasonal biases affecting the validation of biomass estimation models. While collecting ground-truth data is critical, it is often labor-intensive [67], resource-demanding, and logistically challenging, especially in remote and inaccessible wetland regions [58,68].

It is crucial to explicitly consider and maintain a balanced perspective on these inherent uncertainties to overcome limitations, advance methodologies, and enhance the robustness of wetland biomass estimation in similar ecological or bioeconomic studies. Refining ground-truth data collection protocols should be prioritized both before and during sampling in the field. Furthermore, implementing strategies for mitigating spatial and temporal autocorrelation during sampling and model training procedures following [69], along with leveraging data from multiple sensors—such as integrating UAV (Unmanned Aerial Vehicle) observations with satellite remote sensing [70] or combining UAV-LiDAR (Light Detection and Ranging) data with multispectral imagery [71]—as well as improving the temporal scope, will be crucial considerations for future studies. These strategies can provide biomass assessments with increased accuracy and very high spatial resolution, thereby refining ecological and bioeconomic modeling efforts.

## 4. Materials and Methods

### 4.1. Study Area

**Figure 4:**
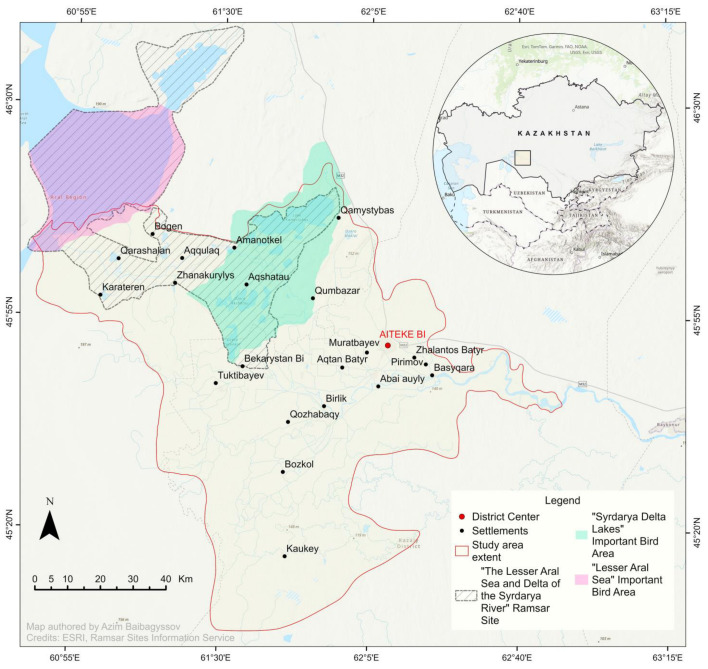
Map of the Syr Darya Delta, Kazakhstan. The study area where biomass mapping took place is outlined in red. WGS 84/UTM zone 41 N (EPSG: 32641).

Our study focuses on the Syr Darya Delta near the northeastern shore of the former Aral Sea in the Kyzylorda Province of Kazakhstan. It is part of the Lesser Aral Sea and Delta of the Syrdarya River Ramsar Site, which the Government of Kazakhstan designated as a Wetland of International Importance in 2012 [72,73]. Within this Ramsar Site, there are two Important Bird Areas (IBAs): the Lesser Aral Sea (144,165 ha) and Syrdarya Delta Lakes (139,400 ha) (as shown in Figure 4).

The current Syr Darya Delta and its adjacent areas are a large sedimentary–alluvial plain with a continental semi-arid to arid climate and annual precipitation below 200 mm [74]. It rests within the Turanian Depression and has a typical slightly undulating lowland terrain, with higher elevation in the north and west and lower elevation in the south. According to Zinabdin [73], the delta stretches about 80 km from the Basykara Dam in the east to the coast of the Northern Aral Sea (NAS) in the west. From north to south, it reaches 120 km in length and is surrounded by sands such as the recently formed Aralkum Desert on the dry bed of the Southern Aral Sea (Great Aral Sea) in the southwest, and the vast Pre-Aral Karakum and Kyzylkum Deserts in the north and the south, respectively.

The source of the Syr Darya Delta waters is the Syr Darya River, the second-largest transboundary river in Central Asia in terms of flow volume, with a catchment area of 219,000 km^2^ [75].

In contrast to the Ili Delta at Lake Balkhash, which has remained largely undisturbed, the Syr Darya Delta at the NAS has undergone considerable changes. In the late 1960s, under the former Soviet Union, it was reclaimed as a large irrigation zone known today as Qazaly (Kazaly), resulting in the development of a network of irrigation canals of various sizes that significantly reshaped the delta’s hydrographic network. This transformation has caused the landscape to lose much of its natural “deltaic” appearance [76], as river branches were cut off from the mainstream or straightened into canals. The irrigation zone, with its area of 15,000 km^2^, has occupied most of the deltaic area and has become one of Kazakhstan’s most important rice cultivation areas, also known for having some of the northernmost rice paddies on the planet [65,77]. However, constructing numerous artificial reservoirs and canals upstream between 1950 and 2017, primarily to supply water for rice and other irrigated cultures such as cotton, has led to reduced river runoff, water shortages downstream, soil salinization, and abandoned land [78,79,80].

Despite the desiccation of the Aral Sea, the environmental and socio-economic situation in the area around the Aral Sea has improved during the last decade, particularly in its northern part. According to Micklin [81], Oskenbayeva [82], and White and Micklin [83], this positive change results from international and local efforts, including the implementation of “Syr Darya control and Northern Aral Sea” (SYNAS) projects. The reconstruction of the Kok-Aral dam and dike complex southwest of the Syr Darya Delta has helped halt the desiccation of the northern part of the Aral Sea. Furthermore, regulatory dams have been established in the delta to ensure that the whole delta, including its lakes and wetlands, now receives more water, especially during the flooding season in spring and early summer [84]. Though the water supply by the Syr Darya River to the delta varies each year, these efforts listed above have positive effects on the wetland ecosystems of the delta, benefiting, among others, the *Phragmites australis* stands [65].

### 4.2. Field Surveying and In Situ Above-Ground and Above-Water Surface Reed Biomass Sampling

This study involved two field survey campaigns in the Syr Darya Delta, with the first conducted in July and August 2019, followed by the second in September and October 2020. Ground-truth data pertaining to land cover, land use, and standing *Phragmites* biomass above ground and above the water surface were collected during both field survey campaigns.

Based on specific ecological and morphological characteristics of the landscape of the Syr Darya Delta, three main investigation areas were selected following an extensive analysis of high-resolution satellite imagery and a comprehensive literature review. These areas, featuring open water, marshes, and meadows characterized by significant reed vegetation, were identified in the following locations of the delta: (a) adjacent to the Kok-Aral dam and dike complex; (b) around deltaic lakes such as Aidarkol and Kotankol next to Bekarystan Bi Village; and (c) along the left branch of the Syr Darya River, next to Tasaryk and Lakaly Villages as well as around the Maryamkol Lake close to Kaukey Village.

**Figure 5:**
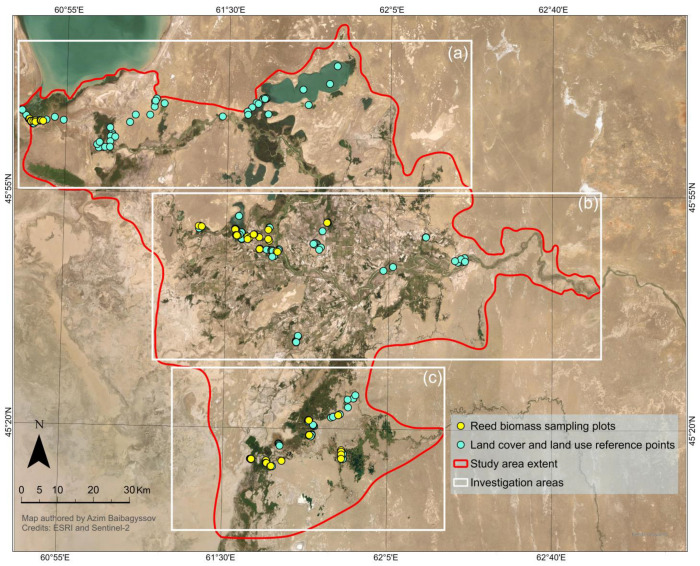
Three main investigation areas (outlined in white rectangles) with collected ground-truth points (*n* = 283) in the Syr Darya Delta, Kazakhstan, during the 2019–2020 field campaigns. (Background: Sentinel-2 scenes from 1 August 2020.)

These areas correspond reasonably well to the Seaside right-bank and left-bank Lake Systems, the Akshatau Lake System, and the Aksai-Kuandarya Lake System of the Syr Darya Delta, as delineated in the work of Kipshakbaev et al. [84]. A map illustrating these investigation areas, along with ground-truth reference points recorded using a handheld Garmin Oregon 750 Global Positioning System (GPS) device (Garmin International Inc., Olathe, KS, USA), which has a reported positioning accuracy of approximately 5 m, is presented in Figure 5.

To ensure the accurate and effective collection of reference ground-truth data, we developed a preliminary sampling scheme based on the concept of stratified random sampling prior to the field campaigns. In this scheme, we identified five primary strata corresponding to different land cover classes: open water, submerged dense reed, non-submerged dense reed vegetation, open reed areas with shrubs, and bare land with open sands (refer to Table 3 for a detailed overview). The stratification was established based on ecological relevance and the variability of habitats supporting common reed’s growth.

Areas representing land cover classes with open water and bare land without reed vegetation were used as grounds for taking reference points, while areas corresponding to land cover classes with reed vegetation were used for biomass sampling. This resulted in a dataset comprising 283 ground-truth points, with 205 related to information on land cover and land use, and 78 representing sampling plots with measured standing above-ground and above-water *Phragmites* biomass, as summarized in Table 4.

For biomass sampling, sites featuring homogeneous reed vegetation of at least 20 m by 20 m were selected for positioning reed biomass sampling plots to ensure conformity with the resolution of the Sentinel-2 images. These plots were marked using a GPS device, and quadrants of 1 m × 1 m size in four to five regular replicates were then spanned over each of the *Phragmites australis*-containing strata to measure the standing biomass of reed after [9]. Therefore, these sampling quadrants covered the range of land cover classes from dense submerged reed *n* = 26 and dense non-submerged reed *n* = 26 to sparse reed mixed with shrub vegetation *n* = 26 (refer to Table 1). To avoid mixed pixels or edge effects, the sampling plots were placed in the centers of large pre-selected sites with homogeneous reed vegetation.

Following [33], we calculated the stem density in each quadrat based on the living stems by counting all living and dead stems. Ten reed stems closest to the diagonal line were randomly chosen and cut 2–3 cm above the ground or water surface, like in the cases of sampling of submerged reeds accomplished by using a boat or by standing in the water in a water-proof wader, depending on the water depth and accessibility. After clipping, the leaves were separated, and stems were chopped into smaller pieces to fit into the oven for drying at 105 °C for 24 h and weighing afterward to determine the dry biomass weight.

The above-ground and above-water surface stand biomass was then calculated by multiplying the average dry weight of one plant—derived from the dry weight of the ten sampled plants—with the previously counted stem density in each sampling quadrant. The resulting yield values were then converted to yields per area of one hectare.

Upon completion of the collection and pre-processing of data, descriptive statistical analysis was performed on *Phragmites australis’s* measured above-ground and above-water surface biomass data.

### 4.3. Satellite Data Acquisition and Spectral Index Calculation

In our study, we used multispectral satellite data from Sentinel-2A and -2B Level-2A, which are part of the Copernicus program by the European Space Agency (ESA). Developed for terrestrial research applications, these satellites provide open-access high-resolution imagery with frequent (3–5 days) revisit times, which makes them particularly valuable for monitoring dynamic ecosystems such as wetlands.

To cover the Syr Darya Delta, we needed four separate Sentinel-2 tiles of 110 × 110 km^2^ each (with footprints IDs: T41TLM, T41TMM, T41TLL, and T41TML). These tiles were used to generate a mosaic of input raster files for each band for every date.

In total, we obtained 24 cloud-free images from the ESA Copernicus Open Access Hub website (the old address: https://scihub.copernicus.eu/; accessed on 18 February 2020 the new address: https://dataspace.copernicus.eu/, accessed on 21 August 2023 ), covering the dates of our field surveying and *Phragmites* biomass sampling in 2019 and 2020. These spaceborne products are provided with orthorectified Bottom-of-Atmosphere (BOA) reflectance, i.e., atmospherically corrected surface reflectance, and ready-to-use imagery in cartographic geometry spanning 13 spectral bands with spatial resolution ranging from 10 to 60 m, as shown in Table 5.

For our modeling, four bands (blue—Band 2; green—Band 3; red—Band 4; and NIR—Band 8) with a spatial resolution of 10 m × 10 m were extracted from each imagery tile and merged into a single mosaic (see Appendix B containing R codes sample as supplementary material for understanding and reproducing the satellite data processing and preparation predictor variables for the RF modeling). These bands were then used to compute two remote sensing indices: NDVI (Equation (1)) and NDWI (Equation (2)). These indices and the four bands have been selected as predictors for reed biomass modeling because they have proven to be suitable predictors for biomass in previous studies, such as Thevs et al. [24], Mogano [85], and Tiškus et al. [86].

We calculated the indices using the following equations:

(1) $$\mathrm{NDVI}=\frac{\rho Nir-\rho Red}{\rho Nir\;+\;\rho Red},$$

(2) $$\mathrm{NDWI}=\frac{\rho Green-\rho Nir}{\rho Green\;+\;\rho Nir}$$

where *ρNir* is the pixel value in the near-infrared band, *ρRed* is the pixel value in the red band, and *ρGreen* is the pixel value in the green band.

In order to exclude the pixels representing settlements and croplands from the modeling process, these areas were identified and manually digitized using the most recent digital cadaster data available on the official state-initiated web portal Qoldau.kz, which monitors agricultural land usage on the territory of Kazakhstan [87,88]. Subsequently, these areas were masked out from the input raster dataset.

These digitization procedures, as well as imagery processing, mosaicking, indices calculations, and other data curations to build prediction models, were carried out using R Studio 2023.06.1 Build 524, QGIS 3.16.14, and ArcGIS Pro 3.2.2.

### 4.4. Overview of the Data and the Study Workflow

**Figure 6:**
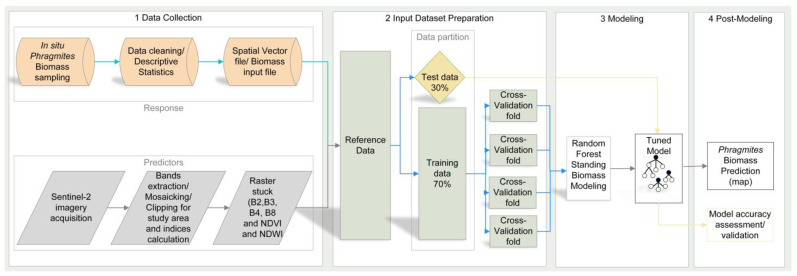
A flowchart illustrating the methodology implemented in this study to map and quantify reed biomass in the wetlands of the Syr Darya Delta, Kazakhstan using multiple Random Forest-based regression models incorporating field measurements of biomass, spectral indices and high-resolution Sentinel-2 satellite imagery (authors’ compilation).

Two types of data were used to conduct this study: reference ground-truth records and high-resolution Sentinel-2 satellite images, which were then combined to assemble the input datasets for the Random Forest (RF) regression modeling, as shown in Figure 6.

In the first step, 78 reed biomass and 205 ground-truth points on the land cover were collected with a stratified sampling technique during field surveying.

In the second step, model input datasets were thoroughly prepared by conflating the dry biomass weight of sampled reed along with the collected points on the land cover as a response variable dataset, and a raster batch of Sentinel-2 spectral channels together with spectral indices compiled for the study area extent as a predictor variables (or “features”) dataset. While the spectral channels included blue (B2), green (B3), red (B4), and near-infrared (B8), the computed indices were the Normalized Difference Vegetation Index (NDVI) and the Normalized Difference Water Index (NDWI).

In the third step, these two input datasets were combined in a machine learning-based regression approach to build and train Random Forest regression models. Predictive reed biomass mapping in the wetlands of the Syr Darya Delta was then carried out using the model with the highest testing accuracy, which was determined through feature selection and hyperparameter tuning after iterative learning of the input variable relationships.

In the fourth step, the predictive performance of the Random Forest regression models, as well as the uncertainty assessment and the quality of the generated map, was evaluated using a ten-fold cross-validation technique.

### 4.5. The Random Forest Modeling, Application, and Performance Assessment

To model a spatially explicit yield map for *Phragmites australis*-dominated wetlands and quantify the area and standing biomass of reed beds in the Syr Darya Delta, Kazakhstan we employed multiple Random Forest regression models, leveraging the strengths of one of the most effective tree-based machine learning algorithms. The selection of Random Forest was driven by its robust modeling capabilities, proficiency in processing complex datasets, and generalization abilities, making it especially an effective method widely used to estimate above-ground biomass under a wide range of biophysical contexts to this day.

Unlike traditional parametric methods, Random Forest does not rely on distribution assumptions of the relationship between the predictors and the response variable [89,90]. This makes it capable of handling outliers and managing noisy and highly correlated predictor variables, thus providing accurate predictions of above-ground biomass with variable importance measures and unbiased error estimates [31]. Additionally, it effectively copes with skewed data distributions, such as in the case of our study.

The algorithm constructs numerous independent decision trees (*ntree*) without pruning using a randomly selected two-thirds training sample from the original dataset by the bootstrapping (multiple random sampling with replacement) method controlled with the node size set by the user [91]. It then tests a random subset of predictor variables at each node in the trees to identify the most efficient split. The final prediction is derived by aggregating and averaging the prediction votes of all the individual trees. The remaining one-third of the original dataset, known as out-of-bag data, stays unseen by the models as an internal validation sample for testing the models’ behavior beyond the data from which they were built. It is then used to assess both model outputs, the mean square error, and node purity.

For our analysis, we built 48 Random Forest above-ground biomass regression models (see Appendix A for more details) using the “rf” function within the “caret” R environment software package v.6.0.94 (see Appendix C containing R codes sample as supplementary material for understanding and reproducing the Random Forest regression modeling) and the Forest-Based Classification and the Regression Tool in ArcGIS Pro 3.2.2. The continuous values of above-ground and above-water surface reed biomass data led us to execute the Random Forest regression models after examining the relationships between reed biomass data and each remote sensing variable (in Section 2.1.).

The optimal parameters were determined with *ntree* set to 1000 and *mtry* calculated as two of the total number of variables and a *minimal node size* of five. This configuration generated the minimized out-of-bag Mean Square Error (MSE) and facilitated the estimation of above-ground and above-water surface biomass of reed on the Syr Darya Delta wetlands using Sentinel-2 images and reed biomass data. To avoid overfitting the models and evaluate their performance and accuracy more precisely, the k-fold cross-validation technique was implemented by setting up the train control for ten folds. This technique involved dividing the data into ten folds or train/test sets so that each point in the dataset occurred exactly once in one of the ten test sets. This allowed us to create a test set that is the same size as the training set but is composed of out-of-sample predictions. Each fold is then randomly assigned to its single test set, avoiding systematic biases in the data.

The assessment of the regression model fit included the computation of the percentage of variance explained by the model, the coefficient of determination (R^2^) (Equation (3)), and the root mean square error (RMSE) (Equation (4)) according to the following equations:

(3) $$R^{2}=1-\frac{\sum_{i=1}^{n}{\left(y_{i}-\hat{y}\right)}^{2}}{\sum_{i=1}^{n}{\left(y_{i}-\hat{y}\right)}^{2}},$$

(4) $$\mathrm{RMSE}=\sqrt{\frac{1}{n}\sum_{i=1}^{n}{\left(y_{i}-\hat{y}\right)}^{2}}$$

where $y_{i}$ is an actual field-measured above-ground and above-water surface biomass value in the *i*th sample, $\hat{y}$ is a predicted above-ground and above-water surface biomass value and $\bar{y}$ represents the mean simulated estimated above-ground and above-water surface biomass for all tested sample points, and n indicates the size of the samples in the different datasets.

## 5. Conclusions

This research work aimed to characterize the resource potential of common reed (*Phragmites australis*) in the Syr Darya Delta, Kazakhstan, through a comprehensive modeling approach utilizing high-resolution Sentinel-2 satellite imagery, spectral indices, and ground-truth above-ground and above-water reed biomass measurements, all applied within multiple Random Forest regression frameworks. The predictive models exhibited high performance, attaining R^2^ values of 0.93 (RMSE = 2.74 t ha^−1^) during calibration and R^2^ = 0.83 (RMSE = 3.71 t ha^−1^) in validation. Models using the Normalized Difference Vegetation Index (NDVI) and Normalized Difference Water Index (NDWI) as key predictors generated precise and spatially explicit yield maps of *Phragmites australis*-dominated wetlands, distinctly outlining the standing biomass of common reed across different types of stands and growing sites. This methodological framework enabled accurate and cost-effective quantification of the reed’s spatial extent and biomass resources.

In the analysis, the total above-ground and above-water biomass of *Phragmites australis* in the study area for 2019–2020 was estimated to be approximately 2.5 million tons. The area of dense reed beds, both submerged and non-submerged, with standing biomass exceeding 10.5 tons per hectare, was calculated to cover 58,935 hectares, representing an estimated biomass of 1,240,789 tons. This underscores the substantial biomass potential inherent in the region’s wetlands.

The practical implications of this research extend beyond the confines of the Syr Darya Delta in Kazakhstan, offering a scalable methodology that can be applied to assess similar wetland ecosystems worldwide. However, while integrating Random Forest regression modeling with high-resolution Sentinel-2 data and in situ biomass samples has proven effective in mapping and quantifying above-ground and above-water biomass in expansive, remote, and dynamic regions like the Syr Darya Delta, characterized by variable hydrological conditions and limited data access, there remains a significant limitation in capturing seasonal dynamics and understanding inter-annual variability in reed biomass due to the short temporal scope of this work.

Future investigations with larger temporal datasets are thus essential for long-term biomass monitoring and understanding their trends. By highlighting these, this study draws attention to further research in tracking biomass dynamics over extended periods and formulating comprehensive and sustainable strategies for managing the distribution and utilization of reed beds, as well as integrating these frequently neglected biomass hotspots into landscape planning initiatives.

## Figures and Tables

**Table 1:** Descriptive statistics of *Phragmites* biomass by relevant reed-containing land cover class in the Syr Darya Delta, Kazakhstan (2019—2020).

Land Cover Class	Number of Samples (n)	Standing Biomass (t ha^−1^)			
		Min	Max	Mean	Standard Deviation
Open reed and shrub vegetation	26	1.14	8.58	4.36	2.46
Non-submerged dense reed	26	7.07	26.01	15.24	5.37
Submerged dense deed	26	6.57	51.33	28.21	11.71

**Table 2:** Characteristics of the predicted above-ground and above-water surface biomass of *Phragmites australis* across various reed-containing land cover classes in the Syr Darya Delta, Kazakhstan (2019—2020), derived from Random Forest regression modeling, high-resolution Sentinel-2 imagery, and ground-truth data.

Land Cover Class	Productivity [t ha^−1^]	Area [ha]	Percentage	Standing Biomass [t]
Open reed and shrub vegetation	0.0–3.5	425,432	75.4	744,506
Non-submerged dense reed	3.5–10.5	79,600	14.1	557,200
	10.5–18.0	27,238	4.8	388,142
Submerged dense reed	18.0–27.4	18,250	3.3	414,275
	27.4–37.8	13,447	2.4	438,372
		Total: 563,967		Total: 2,542,495

**Table 3:** Land cover classes (strata) delineated for the assessment of *Phragmites* biomass in the study area in 2019 and 2020.

Land Cover Class (Strata)	Definition	Photograph
Open water	Open water bodies with <4% vegetation cover	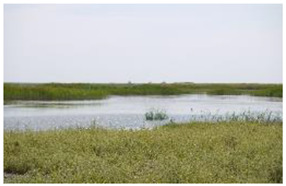
Submerged dense reed	Reed (*Phragmites australis*-dominated) vegetation with a total vegetation cover of 70% or more and submerged soil during most of the year	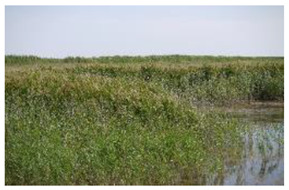
Non-submerged dense reed	Reed (*Phragmites australis*-dominated) vegetation with a total vegetation cover of 70% or more non-submerged soil during most of the year	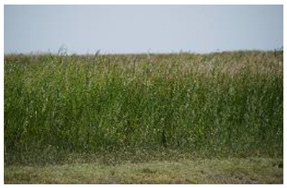
Open reed and shrub vegetation	Reed (*Phragmites australis*-dominated) vegetation, partly interspersed by shrubs with a total vegetation cover of <70%, but at least 20%, and non-submerged soil during most of the year	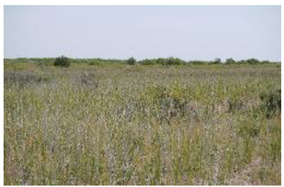
Bare land with open sands	Bare land with open sands with <4% vegetation cover	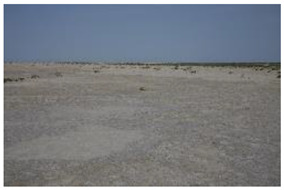

**Table 4:** Summary of ground-truth data collected across the three investigation areas in the Syr Darya Delta, Kazakhstan, during the 2019–2020 field campaigns.

Investigation Areas	Land Cover and Land-Use Points	Reed Biomass Sampling Plots
(a) Wetland areas next to the Kok-Aral dam and dike complex	60	12
(b) Wetland areas around deltaic lakes, such as Aidarkol and Kotankol next to the Bekarystan Bi Village	74	37
(c) Wetland areas along the left branch of the Syr Darya River next to Tasaryk and Lakaly Villages and around the Maryamkol Lake next to the Kaukei Village	71	29
Total	205	78

**Table 5:** Spectral and spatial characteristics of Sentinel-2 imagery data.

Band	Definition	Wavelength (nm)	Spatial Resolution (m)
Band 1	Coastal Aerosol	443	60
Band 2	Blue	490	10
Band 3	Green	560	10
Band 4	Red	665	10
Band 5	Red-edge 1	705	20
Band 6	Red-edge 2	740	20
Band 7	Red-edge 3	783	20
Band 8	Near-infrared (NIR)	842	10
Band 8a	Near-infrared (NIR) narrow	865	20
Band 9	Water vapor	945	60
Band 10	Short-wavelength infrared (SWIR-Cirrus)	1375	60
Band 11	Short-wavelength infrared (SWIR-1)	1610	20
Band 12	Short-wavelength infrared (SWIR-2)	2190	20

## Data Availability

The original contributions presented in this study are included in the article. Further inquiries can be directed to the corresponding author.

## Abbreviations

The following abbreviations are used in this manuscript:

MDPI	Multidisciplinary Digital Publishing Institute
RF	Random Forest
NDVI	Normalized Difference Vegetation Index
NDWI	Normalized Difference Water Index
RMSE	Root mean square error
NIR	Near-infrared
WGS 84	World Geodetic System 1984
UTM	Universal Transverse Mercator
41N	The Northern hemisphere zone 41
GIS	Geographic Information System
EPSG	European Petroleum Survey Group
UAV	Unmanned Aerial Vehicle
LiDAR	Light Detection and Ranging
IBA	Important Bird Area
NAS	North Aral Sea
SYNAS	Syr Darya control and Northern Aral Sea
GPS	Global Positioning System
ESA	European Space Agency
BOA	Bottom-of-Atmosphere

## Appendix A

Table A1 summarizes the detailed characteristics of the 48 Random Forest regression models computed using high-resolution Sentinel-2 satellite data from various dates, two reference datasets, and different combinations of explanatory variables, along with their performance metrics targeted during model calibration.

**Table A1:** Characteristics of 48 RF models and their performance metrics obtained during calibration.

Date	Dataset	Explanatory Variables	RF (No)	% of Variation Explained	Ten-Fold Cross-Validation	
					R^2^	RMSE
13 July 2019	78 biomass data	B2, B3, B4, B8, NDVI and NDWI	rf1	61.2	0.61	8.11
	78 biomass data	NDVI, NDWI, B4 and B2	rf2	59.8	0.59	7.98
	78 biomass data	NDVI and NDWI	rf3	55.4	0.55	8.26
	78 biomass data and 205 land cover points	B2, B3, B4, B8, NDVI and NDWI	rf4	78.5	0.78	4.46
	78 biomass data and 205 land cover points	NDWI, NDVI, B2 and B8	rf5	78.3	0.78	4.53
	78 biomass data and 205 land cover points	NDWI and NDVI	rf6	74.3	0.74	4.94
2 August 2019	78 biomass data	B2, B3, B4, B8, NDVI and NDWI	rf7	61.3	0.61	8.02
	78 biomass data	B4, B2, NDVI and B8	rf8	62.7	0.62	7.78
	78 biomass data	NDVI and NDWI	rf9	55.4	0.55	8.94
	78 biomass data and 205 land cover points	B2, B3, B4, B8, NDVI and NDWI	rf10	69.8	0.69	5.18
	78 biomass data and 205 land cover points	NDVI, NDWI, B2 and B4	rf11	71.2	0.71	5.16
	78 biomass data and 205 land cover points	NDVI and NDWI	rf12	62.6	0.63	6.09
21 September 2019	78 biomass data	B2, B3, B4, B8, NDVI and NDWI	rf13	63.1	0.63	8.19
	78 biomass data	B2, NDVI, B4 and B8	rf14	62.5	0.62	8.24
	78 biomass data	NDVI and NDWI	rf15	42.2	0.42	10.29
	78 biomass data and 205 land cover points	B2, B3, B4, B8, NDVI and NDWI	rf16	69.6	0.69	5.65
	78 biomass data and 205 land cover points	NDVI, NDWI, B2 and B4	rf17	70.9	0.71	5.56
	78 biomass data and 205 land cover points	NDVI and NDWI	rf18	62.5	0.62	6.22
17 July 2020	78 biomass data	B2, B3, B4, B8, NDVI and NDWI	rf19	54.9	0.54	8.90
	78 biomass data	B2, B4, B3 and NDVI	rf20	57.2	0.57	8.75
	78 biomass data	NDVI and NDWI	rf21	34.1	0.34	10.93
	78 biomass data and 205 land cover points	B2, B3, B4, B8, NDVI and NDWI	rf22	71.7	0.72	5.26
	78 biomass data and 205 land cover points	NDWI, NDVI, B2 and B3	rf23	72.1	0.72	5.22
	78 biomass data and 205 land cover points	NDWI and NDVI	rf24	62.8	0.63	6.43
31 August 2020	78 biomass data	B2, B3, B4, B8, NDVI and NDWI	rf25	61.5	0.61	7.75
	78 biomass data	NDVI, NDWI, B4 and B3	rf26	59	0.59	7.96
	78 biomass data	NDVI and NDWI	rf27	60.7	0.60	7.84
	78 biomass data and 205 land cover points	B2, B3, B4, B8, NDVI and NDWI	rf28	67.4	0.67	5.52
	78 biomass data and 205 land cover points	NDWI, NDVI, B4 and B8	rf29	64.9	0.64	5.69
	78 biomass data and 205 land cover points	NDWI and NDVI	rf30	60.6	0.60	5.93
20 September 2020	78 biomass data	B2, B3, B4, B8, NDVI and NDWI	rf31	67.8	0.68	7.29
	78 biomass data	NDVI, NDWI, B4 and B3	rf32	65.3	0.65	7.59
	78 biomass data	NDVI and NDWI	rf33	57.1	0.57	8.69
	78 biomass data and 205 land cover points	B2, B3, B4, B8, NDVI and NDWI	rf34	70.4	0.70	5.24
	78 biomass data and 205 land cover points	NDWI, NDVI, B3 and B2	rf35	65.7	0.66	5.76
	78 biomass data and 205 land cover points	NDWI and NDVI	rf36	60.4	0.60	6.26
Mean 2019	78 biomass data	mean B2, mean B3, mean B4, mean B8, mean NDVI and mean NDWI	rf37	76.6	0.77	6.81
	78 biomass data	mean NDVI, mean NDWI, mean B2 and mean B4	rf38	75.1	0.75	7.04
	78 biomass data	NDVI and NDWI	rf39	65.7	0.66	7.93
	78 biomass data and 205 land cover points	mean B2, mean B3, mean B4, mean B8, mean NDVI and mean NDWI	rf40	80.9	0.81	4.11
	78 biomass data and 205 land cover points	mean NDVI, mean NDWI, mean B2 and mean B8	rf41	81	0.81	4.08
	78 biomass data and 205 land cover points	mean NDVI and mean NDWI	rf42	76.8	0.77	4.46
Mean 2020	78 biomass data	mean B2, mean B3, mean B4, mean B8, mean NDVI and mean NDWI	rf43	66.1	0.66	7.73
	78 biomass data	mean B4, mean NDWI, mean NDVI and mean B3	rf44	67	0.67	7.90
	78 biomass data	mean NDWI and mean NDVI	rf45	66.3	0.66	8.22
	78 biomass data and 205 land cover points	mean B2, mean B3, mean B4, mean B8, mean NDVI and mean NDWI	rf46	76.9	0.77	4.60
	78 biomass data and 205 land cover points	mean NDWI, mean NDVI mean B4 and mean B8	rf47	74.7	0.75	4.86
	78 biomass data and 205 land cover points	mean NDVI and mean NDWI	rf48	72.7	0.72	5.05

## Appendix B

Sample of R codes for understanding and reproducing the high-resolution Sentinel-2 satellite band extraction, mosaicking, and preparation as predictor variables for the Random Forest regression modeling.

######Preparing high-resolution bands of Sentinel-2 from 20092020#####

# Setting the working directory

setwd(“C:/All data/20200920”)

getwd()

#installing and loading required r-packages

library(raster)

library(rgdal)

library(terra)

########################----b02----###########################################

###Reading the jp2 files for band2 (TLL, TML, TLM and TMM)

jp2.TLL.b02 <- readGDAL(“S2B_MSIL2A_20200920T064629_N0500_R020_T41TLL_20230504T083232.SAFE/GRANULE/L2A_T41TLL_A018493_20200920T064720/IMG_DATA/R10m/T41TLL_20200920T064629_B02_10m.jp2”)

jp2.TML.b02 <- readGDAL(“S2B_MSIL2A_20200920T064629_N0500_R020_T41TML_20230504T083232.SAFE/GRANULE/L2A_T41TML_A018493_20200920T064720/IMG_DATA/R10m/T41TML_20200920T064629_B02_10m.jp2”)

jp2.TLM.b02 <- readGDAL(“S2B_MSIL2A_20200920T064629_N0500_R020_T41TLM_20230504T083232.SAFE/GRANULE/L2A_T41TLM_A018493_20200920T064720/IMG_DATA/R10m/T41TLM_20200920T064629_B02_10m.jp2”)

jp2.TMM.b02 <- readGDAL(“S2B_MSIL2A_20200920T064629_N0500_R020_T41TMM_20230504T083232.SAFE/GRANULE/L2A_T41TMM_A018493_20200920T064720/IMG_DATA/R10m/T41TMM_20200920T064629_B02_10m.jp2”)

###Writing each as a TIFF file

writeGDAL(jp2.TLL.b02,”TLL.b02.20.09.tif”, drivername = “GTiff”, type = “Int32”,

mvFlag = NA, options = NULL, copy_drivername = “GTiff”, setStatistics = FALSE,

colorTables = NULL, catNames = NULL)

writeGDAL(jp2.TML.b02,”TML.b02.20.09.tif”, drivername = “GTiff”, type = “Int32”,

mvFlag = NA, options = NULL, copy_drivername = “GTiff”, setStatistics = FALSE,

colorTables = NULL, catNames = NULL)

writeGDAL(jp2.TLM.b02,”TLM.b02.20.09.tif”, drivername = “GTiff”, type = “Int32”,

mvFlag = NA, options = NULL, copy_drivername = “GTiff”, setStatistics = FALSE,

colorTables = NULL, catNames = NULL)

writeGDAL(jp2.TMM.b02,”TMM.b02.20.09.tif”, drivername = “GTiff”, type = “Int32”,

mvFlag = NA, options = NULL, copy_drivername = “GTiff”, setStatistics = FALSE,

colorTables = NULL, catNames = NULL)

###Loading the 4 tiles of band2

TLL_200920_b02 <- raster(“TLL.b02.20.09.tif”)

TLM_200920_b02 <- raster(“TLM.b02.20.09.tif”)

TML_200920_b02 <- raster(“TML.b02.20.09.tif”)

TMM_200920_b02 <- raster(“TMM.b02.20.09.tif”)

###Dividing each by 10000 (Quantification value) to get reflectance values (Float)

TLL_200920_b02 <- TLL_200920_b02/10000

TLM_200920_b02 <- TLM_200920_b02/10000

TML_200920_b02 <- TML_200920_b02/10000

TMM_200920_b02 <- TMM_200920_b02/10000

###Mosaicking the 4 tiles of band2

#Changing the names of TLMB to the layer names of TMMB

names(TLM_200920_b02) <- names(TMM_200920_b02)

#Making a mosaick of the two (TLM and TMM) tiles

TLM_TMM_20.09.20b02 <- mosaic(TMM_200920_b02,TLM_200920_b02, fun = mean)

#adding the third (TML) tile

names(TML_200920_b02) <-names(TLM_TMM_20.09.20b02)

TLM_TMM_TML_20.09.20b02 <- mosaic(TML_200920_b02,TLM_TMM_20.09.20b02, fun = mean)

#adding the fourth (TLL) tile

names(TLL_200920_b02) <- names(TLM_TMM_TML_20.09.20b02)

TLM_TMM_TML_TLL_20.09.20b02 <- mosaic(TLL_200920_b02,TLM_TMM_TML_20.09.20b02, fun = mean)

#checking on the mosaicking output by plotting

plot(TLM_TMM_TML_TLL_20.09.20b02)

###Writing a mosaic of band2 as a TIFF file

writeRaster(TLM_TMM_TML_TLL_20.09.20b02, ”B2_200920.tif”)

########################----b03----###########################################

###Reading the jp2 files for band3 (TLL,TML,TLM and TMM)

jp2.TLL.b03 <- readGDAL(“S2B_MSIL2A_20200920T064629_N0500_R020_T41TLL_20230504T083232.SAFE/GRANULE/L2A_T41TLL_A018493_20200920T064720/IMG_DATA/R10m/T41TLL_20200920T064629_b03_10m.jp2”)

jp2.TML.b03 <- readGDAL(“S2B_MSIL2A_20200920T064629_N0500_R020_T41TML_20230504T083232.SAFE/GRANULE/L2A_T41TML_A018493_20200920T064720/IMG_DATA/R10m/T41TML_20200920T064629_b03_10m.jp2”)

jp2.TLM.b03 <- readGDAL(“S2B_MSIL2A_20200920T064629_N0500_R020_T41TLM_20230504T083232.SAFE/GRANULE/L2A_T41TLM_A018493_20200920T064720/IMG_DATA/R10m/T41TLM_20200920T064629_b03_10m.jp2”)

jp2.TMM.b03 <- readGDAL(“S2B_MSIL2A_20200920T064629_N0500_R020_T41TMM_20230504T083232.SAFE/GRANULE/L2A_T41TMM_A018493_20200920T064720/IMG_DATA/R10m/T41TMM_20200920T064629_b03_10m.jp2”)

###Writing each as a TIFF file

writeGDAL(jp2.TLL.b03,”TLL.b03.20.09.tif”, drivername = “GTiff”, type = “Int32”,

mvFlag = NA, options = NULL, copy_drivername = “GTiff”, setStatistics = FALSE,

colorTables = NULL, catNames = NULL)

writeGDAL(jp2.TML.b03,”TML.b03.20.09.tif”, drivername = “GTiff”, type = “Int32”,

mvFlag = NA, options = NULL, copy_drivername = “GTiff”, setStatistics = FALSE,

colorTables = NULL, catNames = NULL)

writeGDAL(jp2.TLM.b03,”TLM.b03.20.09.tif”, drivername = “GTiff”, type = “Int32”,

mvFlag = NA, options = NULL, copy_drivername = “GTiff”, setStatistics = FALSE,

colorTables = NULL, catNames = NULL)

writeGDAL(jp2.TMM.b03,”TMM.b03.20.09.tif”, drivername = “GTiff”, type = “Int32”,

mvFlag = NA, options = NULL, copy_drivername = “GTiff”, setStatistics = FALSE,

colorTables = NULL, catNames = NULL)

###Loading the 4 tiles of band3

TLL_200920_b03 <- raster(“TLL.b03.20.09.tif”)

TLM_200920_b03 <- raster(“TLM.b03.20.09.tif”)

TML_200920_b03 <- raster(“TML.b03.20.09.tif”)

TMM_200920_b03 <- raster(“TMM.b03.20.09.tif”)

###Dividing each by 10000 (Quantification value) to get reflectance values (Float)

TLL_200920_b03 <- TLL_200920_b03/10000

TLM_200920_b03 <- TLM_200920_b03/10000

TML_200920_b03 <- TML_200920_b03/10000

TMM_200920_b03 <- TMM_200920_b03/10000

###Mosaicking the 4 tiles of band2

#Changing the names of TLMB to the layer names of TMMB

names(TLM_200920_b03) <- names(TMM_200920_b03)

#Making a mosaick of the two (TLM and TMM) tiles

TLM_TMM_20.09.20b03 <- mosaic(TMM_200920_b03,TLM_200920_b03, fun = mean)

plot(TLM_TMM_20.09.20b03)

#adding the third (TML) tile

names(TML_200920_b03) <- names(TLM_TMM_20.09.20b03)

TLM_TMM_TML_20.09.20b03 <- mosaic(TML_200920_b03,TLM_TMM_20.09.20b03, fun = mean)

#adding the fourth (TLL) tile

names(TLL_200920_b03) <- names(TLM_TMM_TML_20.09.20b03)

TLM_TMM_TML_TLL_20.09.20b03 <- mosaic(TLL_200920_b03,TLM_TMM_TML_20.09.20b03, fun = mean)

#checking on the mosaicking output by plotting

plot(TLM_TMM_TML_TLL_20.09.20b03)

###Writing a mosaic of band3 as a TIFF file

writeRaster(TLM_TMM_TML_TLL_20.09.20b03, ”B3_200920.tif”)

########################----b04----###########################################

###Reading the jp2 files for band4 (TLL,TML,TLM and TMM)

jp2.TLL.b04 <- readGDAL(“S2B_MSIL2A_20200920T064629_N0500_R020_T41TLL_20230504T083232.SAFE/GRANULE/L2A_T41TLL_A018493_20200920T064720/IMG_DATA/R10m/T41TLL_20200920T064629_b04_10m.jp2”)

jp2.TML.b04 <- readGDAL(“S2B_MSIL2A_20200920T064629_N0500_R020_T41TML_20230504T083232.SAFE/GRANULE/L2A_T41TML_A018493_20200920T064720/IMG_DATA/R10m/T41TML_20200920T064629_b04_10m.jp2”)

jp2.TLM.b04 <- readGDAL(“S2B_MSIL2A_20200920T064629_N0500_R020_T41TLM_20230504T083232.SAFE/GRANULE/L2A_T41TLM_A018493_20200920T064720/IMG_DATA/R10m/T41TLM_20200920T064629_b04_10m.jp2”)

jp2.TMM.b04 <- readGDAL(“S2B_MSIL2A_20200920T064629_N0500_R020_T41TMM_20230504T083232.SAFE/GRANULE/L2A_T41TMM_A018493_20200920T064720/IMG_DATA/R10m/T41TMM_20200920T064629_b04_10m.jp2”)

###Writing each as a TIFF file

writeGDAL(jp2.TLL.b04,”TLL.b04.20.09.tif”, drivername = “GTiff”, type = “Int32”,

mvFlag = NA, options = NULL, copy_drivername = “GTiff”, setStatistics = FALSE,

colorTables = NULL, catNames = NULL)

writeGDAL(jp2.TML.b04,”TML.b04.20.09.tif”, drivername = “GTiff”, type = “Int32”,

mvFlag = NA, options = NULL, copy_drivername = “GTiff”, setStatistics = FALSE,

colorTables = NULL, catNames = NULL)

writeGDAL(jp2.TLM.b04,”TLM.b04.20.09.tif”, drivername = “GTiff”, type = “Int32”,

mvFlag = NA, options = NULL, copy_drivername = “GTiff”, setStatistics = FALSE,

colorTables = NULL, catNames = NULL)

writeGDAL(jp2.TMM.b04,”TMM.b04.20.09.tif”, drivername = “GTiff”, type = “Int32”,

mvFlag = NA, options = NULL, copy_drivername = “GTiff”, setStatistics = FALSE,

colorTables = NULL, catNames = NULL)

###Loading the 4 tiles of band4

TLL_200920_b04 <- raster(“TLL.b04.20.09.tif”)

TLM_200920_b04 <- raster(“TLM.b04.20.09.tif”)

TML_200920_b04 <- raster(“TML.b04.20.09.tif”)

TMM_200920_b04 <- raster(“TMM.b04.20.09.tif”)

###Dividing each by 10000 (Quantification value) to get reflectance values (Float)

TLL_200920_b04 <- TLL_200920_b04/10000

TLM_200920_b04 <- TLM_200920_b04/10000

TML_200920_b04 <- TML_200920_b04/10000

TMM_200920_b04 <- TMM_200920_b04/10000

###Mosaicking the 4 tiles of band4

#Changing the names of TLMB to the layer names of TMMB

names(TLM_200920_b04) <- names(TMM_200920_b04)

#Making a mosaick of the two (TLM and TMM) tiles

TLM_TMM_20.09.20b04 <- mosaic(TMM_200920_b04,TLM_200920_b04, fun = mean)

#adding the third (TML) tile

names(TML_200920_b04) <- names(TLM_TMM_20.09.20b04)

TLM_TMM_TML_20.09.20b04<-mosaic(TML_200920_b04,TLM_TMM_20.09.20b04, fun = mean)

#adding the fourth (TLL) tile

names(TLL_200920_b04) <- names(TLM_TMM_TML_20.09.20b04)

TLM_TMM_TML_TLL_20.09.20b04 <- mosaic(TLL_200920_b04,TLM_TMM_TML_20.09.20b04, fun = mean)

#checking on the mosaicking output by plotting

plot(TLM_TMM_TML_TLL_20.09.20b04)

###Writing a mosaic of band4 as a TIFF file

writeRaster(TLM_TMM_TML_TLL_20.09.20b04, ”B4_200920.tif”)

########################----b08----###########################################

###Reading the jp2 files for band8 (TLL,TML,TLM and TMM)

jp2.TLL.b08 <- readGDAL(“S2B_MSIL2A_20200920T064629_N0500_R020_T41TLL_20230504T083232.SAFE/GRANULE/L2A_T41TLL_A018493_20200920T064720/IMG_DATA/R10m/T41TLL_20200920T064629_b08_10m.jp2”)

jp2.TML.b08 <- readGDAL(“S2B_MSIL2A_20200920T064629_N0500_R020_T41TML_20230504T083232.SAFE/GRANULE/L2A_T41TML_A018493_20200920T064720/IMG_DATA/R10m/T41TML_20200920T064629_b08_10m.jp2”)

jp2.TLM.b08 <- readGDAL(“S2B_MSIL2A_20200920T064629_N0500_R020_T41TLM_20230504T083232.SAFE/GRANULE/L2A_T41TLM_A018493_20200920T064720/IMG_DATA/R10m/T41TLM_20200920T064629_b08_10m.jp2”)

jp2.TMM.b08 <- readGDAL(“S2B_MSIL2A_20200920T064629_N0500_R020_T41TMM_20230504T083232.SAFE/GRANULE/L2A_T41TMM_A018493_20200920T064720/IMG_DATA/R10m/T41TMM_20200920T064629_b08_10m.jp2”)

###Writing each as a TIFF file

writeGDAL(jp2.TLL.b08,”TLL.b08.20.09.tif”, drivername = “GTiff”, type = “Int32”,

mvFlag = NA, options = NULL, copy_drivername = “GTiff”, setStatistics = FALSE,

colorTables = NULL, catNames = NULL)

writeGDAL(jp2.TML.b08,”TML.b08.20.09.tif”, drivername = “GTiff”, type = “Int32”,

mvFlag = NA, options = NULL, copy_drivername = “GTiff”, setStatistics = FALSE,

colorTables = NULL, catNames = NULL)

writeGDAL(jp2.TLM.b08,”TLM.b08.20.09.tif”, drivername = “GTiff”, type = “Int32”,

mvFlag = NA, options = NULL, copy_drivername = “GTiff”, setStatistics = FALSE,

colorTables = NULL, catNames = NULL)

writeGDAL(jp2.TMM.b08,”TMM.b08.20.09.tif”, drivername = “GTiff”, type = “Int32”,

mvFlag = NA, options = NULL, copy_drivername = “GTiff”, setStatistics = FALSE,

colorTables = NULL, catNames = NULL)

###Loading the 4 tiles of band8

TLL_200920_b08 <- raster(“TLL.b08.20.09.tif”)

TLM_200920_b08 <- raster(“TLM.b08.20.09.tif”)

TML_200920_b08 <- raster(“TML.b08.20.09.tif”)

TMM_200920_b08 <- raster(“TMM.b08.20.09.tif”)

###Dividing each by 10000 (Quantification value) to get reflectance values (Float)

TLL_200920_b08 <- TLL_200920_b08/10000

TLM_200920_b08 <- TLM_200920_b08/10000

TML_200920_b08 <- TML_200920_b08/10000

TMM_200920_b08 <- TMM_200920_b08/10000

###Mosaicking the 4 tiles of band8

#Changing the names of TLMB to the layer names of TMMB

names(TLM_200920_b08) <- names(TMM_200920_b08)

#Making a mosaick of the two (TLM and TMM) tiles

TLM_TMM_20.09.20b08 <- mosaic(TMM_200920_b08,TLM_200920_b08, fun = mean)

#adding the third (TML) tile

names(TML_200920_b08) <- names(TLM_TMM_20.09.20b08)

TLM_TMM_TML_20.09.20b08 <- mosaic(TML_200920_b08,TLM_TMM_20.09.20b08, fun = mean)

#adding the fourth (TLL) tile

names(TLL_200920_b08) <- names(TLM_TMM_TML_20.09.20b08)

TLM_TMM_TML_TLL_20.09.20b08 <- mosaic(TLL_200920_b08,TLM_TMM_TML_20.09.20b08, fun = mean)

#checking on the mosaicking output by plotting

plot(TLM_TMM_TML_TLL_20.09.20b08)

###Writing a mosaic of band8 as a TIFF file

writeRaster(TLM_TMM_TML_TLL_20.09.20b08, ”B8_200920.tif”)

#############################################################################

###reading the ready rasters of the bands

band2 <- raster(“B2_200920.tif”)

band3 <- raster(“B3_200920.tif”)

band4 <- raster(“B4_200920.tif”)

band8 <- raster(“B8_200920.tif”)

#############################################################################

### clipping rasters of the bands to study region extent

## Loading the shapefile of the Syr Darya Delta

Delta_ext = readOGR(dsn = “C:/All data”, layer = “AOIandMask”)

SyrD_mask = readOGR(dsn = “C:/All data”, layer = “AOIwithholes”)

# Cropping and masking rasters of the bands to the Syr Darya Delta without irrigated croplands

delt_cropb02 <- crop(band2, Delta_ext)

delt_aoib02 <- mask(delt_cropb02, SyrD_mask)

delt_cropb03 <- crop(band3, Delta_ext)

delt_aoib03 <- mask(delt_cropb03, SyrD_mask)

delt_cropb04 <- crop(band4, Delta_ext)

delt_aoib04 <- mask(delt_cropb04, SyrD_mask)

delt_cropb08 <- crop(band8, Delta_ext)

delt_aoib08 <- mask(delt_cropb08, SyrD_mask)

#Writing each cropped and masked rasters as a TIFF file

writeRaster(delt_aoib02,”mb2_200920.tif”)

writeRaster(delt_aoib03,”mb3_200920.tif”)

writeRaster(delt_aoib04,”mb4_200920.tif”)

writeRaster(delt_aoib08,”mb8_200920.tif”)

### Calculating NDVI and NDWI indices and writing them as TIFF files

ndvi200920 <- (delt_aoib08 − delt_aoib04)/(delt_aoib08 + delt_aoib04)

writeRaster(ndvi200920,”ndvi200920m.tif”)

ndwi200920 <- (delt_aoib03 − delt_aoib08)/(delt_aoib03 + delt_aoib08)

writeRaster(ndwi200920,”ndwi200920m.tif”)

## Appendix C

R code sample of establishing the Random Forest above-ground biomass regression models by setting up the train control for ten-fold cross-validation using the “rf” function within the “caret” R environment software package.

############### Title: Establishing Random Forest regression models of reed above-ground and above-water biomass in the Syr Darya Delta, Kazakhstan using high-resolution Sentinel-2 images and ground-truth biomass data (2019–2020)

### Setting the working directory

setwd(“C:/All data”)

### Checking the correctness of working directory

getwd()

### Changing the default Russian language of arguments in Console to English

Sys.setenv(LANG = “en”)

### Removing my working environment

rm(list = ls())

### Installing required packages and downloading their libraries

library(sp)

library(raster)

library(sf)

library(rgdal)

library(terra)

library(randomForest)

library(ggplot2)

library(MASS)

library(caret)

###Loading and exploring the high-resolution Sentinel-2 data for September 20 2020

b2_200920 <- raster(“mb2_200920.tif”)

b3_200920 <- raster(“mb3_200920.tif”)

b4_200920 <- raster(“mb4_200920.tif”)

b8_200920 <- raster(“mb8_200920.tif”)

NDVI_200920 <- raster(“ndvi200920m.tif”)

NDWI_200920 <- raster(“ndwi200920m.tif”)

### Creating a rasterstack from all bands, NDVI and NDWI indices

Sen2_200920 <- stack(b2_200920,b3_200920,b4_200920,b8_200920,NDVI_200920,NDWI_200920)

### Examining the stack and plotting it

Sen2_200920

names(Sen2_200920)

st_crs(Sen2_200920)

plot(Sen2_200920)

#### Loading shape-files with ground-truth data points

#78 gpspoints

Points_78 = readOGR(dsn = “C:/All data”, layer = “bio_78_points”)

#283 gpspoints

Points_283 = readOGR(dsn = “C:/All data”, layer = “bio_283_points”)

### Examining and plotting them

st_crs(Points_78)

st_crs(Points_283)

plot(Points_78)

plot(Points_283)

###Extracting values from rasters to the points locations and making data frame

# for 20092020

pointVals78_200920 <- extract(Sen2_200920, Points_78)

pointVals283_200920 <- extract(Sen2_200920, Points_283)

# Printing the object type to answer the question above

is(pointVals78_200920)

is(pointVals283_200920)

# Binding the extracted values to the biomass plot data table to make a data frame with values and metadata

DF78_200920 <- cbind(data.frame(Points_78), pointVals78_200920)

DF283_200920 <- cbind(data.frame(Points_283), pointVals283_200920)

#inspecting data frames

head(DF78_200920)

names(DF78_200920)

head(DF283_200920)

names(DF283_200920)

# removing unnecessary data fields

DF78_200920$id <- NULL

DF78_200920$Name <- NULL

DF78_200920$coords.x1 <- NULL

DF78_200920$coords.x2 <- NULL

DF78_200920$optional <- NULL

DF283_200920$id <- NULL

DF283_200920$Name <- NULL

DF283_200920$coords.x1 <- NULL

DF283_200920$coords.x2 <- NULL

DF283_200920$optional <- NULL

########################RF-modeling#########################################

###Defining control for 10-fold cross-validation

control <- trainControl(method = “cv”, number = 10, summaryFunction = defaultSummary)

###Training the Random Forest regression models for high-resolution Sentinel-2 data from September 20 2020

#rf31 for 78 points September 20 2020

set.seed (1234)

rf31 <- train(Biomass ~ mb2_200920 + mb3_200920 + mb4_200920 + mb8_200920 + ndvi200920m + ndwi200920m,

data = DF78_200920, method = “rf”, trControl = control, na.action = na.omit, importance = TRUE)

#Printing model results

print(rf31)

#Extracting performance metrics

res_rf31 <- rf31$results

print(res_rf31)

#Calculating the percentage of variance explained

variance_explained_percentage_rf31 <- res_rf31$Rsquared * 100

print(variance_explained_percentage_rf31)

#Obtaining importance of Variables

var_importance_rf31 <- varImp(rf31, scale = FALSE)

print(var_importance_rf31)

#rf32 for 78 points September 20 2020

set.seed (1234)

rf32 <- train(Biomass ~ ndvi200920m + ndwi200920m + mb4_200920 + mb3_200920,

data = DF78_200920, method = “rf”, trControl = control, na.action = na.omit, importance = TRUE)

#Printing model results

print(rf32)

#Extracting performance metrics

res_rf32 <- rf32$results

print(res_rf32)

#Calculating the percentage of variance explained

variance_explained_percentage_rf32 <- res_rf32$Rsquared * 100

print(variance_explained_percentage_rf32)

#Obtaining importance of Variables

var_importance_rf32 <- varImp(rf32, scale = FALSE)

print(var_importance_rf32)

#rf33 for 78 points September 20 2020

set.seed (1234)

rf33 <- train(Biomass ~ ndvi200920m + ndwi200920m,

data = DF78_200920, method = “rf”, trControl = control, na.action = na.omit, importance = TRUE)

#Printing model results

print(rf33)

#Extracting performance metrics

res_rf33 <- rf33$results

print(res_rf33)

#Calculating the percentage of variance explained

variance_explained_percentage_rf33 <- res_rf33$Rsquared * 100

print(variance_explained_percentage_rf33)

#Obtaining importance of Variables

var_importance_rf33 <- varImp(rf33, scale = FALSE)

print(var_importance_rf33)

###rf34 for 283 points September 20 2020

set.seed (1234)

rf34 <- train(Biomass ~ mb2_200920 + mb3_200920 + mb4_200920 + mb8_200920 + ndvi200920m + ndwi200920m,

data = DF283_200920, method = “rf”, trControl = control, na.action = na.omit, importance = TRUE)

#Printing model results

print(rf34)

#Extracting performance metrics

res_rf34 <- rf34$results

print(res_rf34)

#Calculating the percentage of variance explained

variance_explained_percentage_rf34 <- res_rf34$Rsquared * 100

print(variance_explained_percentage_rf34)

#Obtaining importance of Variables

var_importance_rf34 <- varImp(rf34, scale = FALSE)

print(var_importance_rf34)

#rf35 for 283 points September 20 2020

set.seed (1234)

rf35 <- train(Biomass ~ ndwi200920m + ndvi200920m + mb3_200920 + mb2_200920,

data = DF283_200920, method = “rf”, trControl = control, na.action = na.omit, importance = TRUE)

#Printing model results

print(rf35)

#Extracting performance metrics

res_rf35 <- rf35$results

print(res_rf35)

#Calculating the percentage of variance explained

variance_explained_percentage_rf35 <- res_rf35$Rsquared * 100

print(variance_explained_percentage_rf35)

#Obtaining importance of Variables

var_importance_rf35 <- varImp(rf35, scale = FALSE)

print(var_importance_rf35)

#rf36 for 283 points September 20 2020

set.seed (1234)

rf36 <- train(Biomass ~ ndwi200920m + ndvi200920m,

data = DF283_200920, method = “rf”, trControl = control, na.action = na.omit, importance = TRUE)

#Printing model results

print(rf36)

#Extracting performance metrics

res_rf36 <- rf36$results

print(res_rf36)

#Calculating the percentage of variance explained

variance_explained_percentage_rf36 <- res_rf36$Rsquared * 100

print(variance_explained_percentage_rf36)

#Obtaining importance of Variables

var_importance_rf36 <- varImp(rf36, scale = FALSE)

print(var_importance_rf36).
